# How antidepressant drugs act: A primer on neuroplasticity as the eventual mediator of antidepressant efficacy

**DOI:** 10.4103/0019-5545.74318

**Published:** 2010

**Authors:** Chittaranjan Andrade, N. Sanjay Kumar Rao

**Affiliations:** Department of Psychopharmacology, National Institute of Mental Health and Neurosciences, Bangalore, India; 1 The Logos Centre for Cognitive Behavioural Therapy and Mental Health Promotion, Tees Esk and Wear Valley NHS Trust, County Hospital, Durham, UK

**Keywords:** Depression, stress, antidepressant drugs, electroconvulsive therapy, hippocampus, amygdala, prefrontal cortex, neuroplasticity, mechanism of action

## Abstract

Depression is conventionally viewed as a state of chemical imbalance, and antidepressants are suggested to act through increasing monoaminergic neurotransmission. These views are currently considered simplistic. This article examines the animal and human literature on the neurohistological mechanisms underlying stress, depression and antidepressant treatment. Pathological stress and depression are associated with changes such as loss of dendritic spines, shrinkage of the dendritic tree and loss of synapses in the hippocampus and prefrontal cortex. There is also a decrease in glia. Apoptosis may occur under extreme circumstances. In contrast, there is increased dendritic arborization and synaptogenesis in the amygdala. Antidepressant treatment protects against and even reverses some but not all of these stress-induced neurohistological changes. Pathological stress results in an aberrant neuroplasticity response characterized by abnormally increased activity in the amygdala and by impaired functioning of the hippocampus, prefrontal cortex and downstream structures. This aberrant neuroplasticity response directly explains most of the clinical symptoms of depression. Antidepressant treatment protects against stress-induced pathoplastic neurohistological and neurocognitive changes. Antidepressant treatment also restores functional neuroplasticity in stressed organisms and, thereby, presumably, facilitates re-adaptation through learning and memory mechanisms. Thus, the stress–depression syndrome and the therapeutic and prophylactic efficacy of antidepressant treatments can be explained through a hardwiring analogy. In this context, glutamate is an important neurotransmitter.

## INTRODUCTION

There is a very large body of evidence which, when put together, suggests that antidepressant treatments act by inducing neuroplastic changes in the brain. This article provides a simple (and very much simplified!) explanation of the neuroplasticity hypothesis of antidepressant action.

### How do antidepressant drugs act?

Conventional answers are basically based on premises such as “depression is an insufficiency of neurotransmitters” and “depression is a state of chemical imbalance in the brain”; antidepressants act by “replenishing the neurotransmitters” or “correcting the chemical imbalance.” Such answers are incomplete and, in the light of present knowledge, possibly even obsolete.

### Conventional explanations

The conventional explanations of antidepressant mechanisms merely describe a proximal action of antidepressant drugs; some examples follow:[1]

1. Antidepressant drugs inhibit the reuptake of monoamines (such as serotonin, noradrenaline and dopamine) into the presynaptic neuron; persistence of these monoamines in the synaptic cleft results in increased postsynaptic receptor stimulation and hence in increased postsynaptic neurotransmission. This effect, presumably, corrects or compensates for the neurophysiological deficits that underlie depression. Examples of drugs that inhibit monoamine reuptake are the tricyclic antidepressants (TCA), the selective serotonin reuptake inhibitors (SSRIs), the serotonin–norepinephrine reuptake inhibitors (SNRIs) and others.
2. Antidepressant drugs inhibit the breakdown of monoamines (such as serotonin, noradrenaline and dopamine) in the storage vesicles of the presynaptic neuron. Preservation of these monoamines presumably improves the efficiency of synaptic neurotransmission. This may correct or compensate for the neurophysiological deficits that underlie depression. Drugs that inhibit monoamine breakdown are the monoamine oxidase inhibitors (MAOIs).
3. Antidepressant drugs increase the reuptake of serotonin (tianeptine), increase the release of serotonin and norepinephrine (mirtazapine), act directly on serotonin and melatonin receptors (agomelatine) or otherwise influence synaptic neurotransmission. With the exception of agomelatine, the focus of explanatory mechanisms is on the great triumvirate: serotonin, norepinephrine and dopamine.

Mechanisms have also been proposed for somatic treatments with antidepressant action. These treatments include electroconvulsive therapy (ECT), repetitive transcranial magnetic stimulation (rTMS), vagus nerve stimulation (VNS), transcranial direct current stimulation (tDCS), light therapy, sleep deprivation therapy and others. The focus of the present article is on the pharmacological and not on the somatic treatments.

### Limitations of synaptic explanations for antidepressant action

A fundamental problem with the synaptic explanations described above is that, while they are immediate, the antidepressant response is delayed. For example, the TCA inhibit the reuptake of monoamines as soon as they are absorbed into the body and transported across the blood-brain barrier, i.e., within hours of administration. However, the antidepressant response takes weeks or longer to develop and complete and, hence, for response and remission to occur. Therefore, to say that the TCA act by increasing the presence of neurotransmitters in the synapse is about as complete as saying that cars run because of an increased presence of fuel in the tank. Clearly, just as mechanisms downstream to the presence of fuel in the tank enable a car to move, so too must there be something downstream to increase the synaptic availability of monoamines to fully explain antidepressant mechanisms.

### Extended explanations

Extended explanations have been offered.[1,2] For example, it is suggested that the greater availability of neurotransmitters in the synapse results in compensatory downregulation of monoamine receptors. Downregulation of the (facilitatory) postsynaptic receptors decreases the activity of the postsynaptic neurons. Downregulation of the (inhibitory) presynaptic receptors increases the activity of the presynaptic neurons. These changes develop across days to weeks, presumably restore the balance of neuronal activity and, presumably, explain the delay in the onset of action of antidepressant drugs. Examples of such extended explanations are:

- TCA downregulate the presynaptic alpha-2 adrenoceptors and the postsynaptic beta adrenoceptors.
- SSRIs downregulate the presynaptic 5-HT1a serotonergic receptors and the postsynaptic 5-HT2 serotonergic receptors.

Such extended explanations are still incomplete. All that they add to the original model, to use the metaphor of the car, is to invoke the actions of the accelerator and brakes. We still need to know what happens beneath the bonnet, i.e., how the engine runs. This is where neuroplasticity comes in.

## THE NEUROPLASTICITY HYPOTHESIS IN A NUTSHELL

The neuroplasticity hypothesis of antidepressant action suggests that specific, dysfunctional histological changes in the hippocampus, prefrontal cortex, amygdala and other parts of the brain explain the clinical features of depression. In other words, depression is a disorder of the hardwiring of the brain, and not a state of chemical imbalance. Antidepressants act by protecting against and reversing at least some of these neurohistological changes.[3] These changes are discussed at greater length in later sections.

## FUNDAMENTAL ASSUMPTIONS OF THE NEUROPLASTICITY HYPOTHESIS

### 1. The neurobiology of depression can be understood from the effects of stress

It has long been known that stress triggers and maintains the state of depression. Learning how to cope better with stress has both therapeutic and prophylactic antidepressant actions, as is evident from studies on cognitive behavior therapy (CBT). Therefore, whatever stress does to the brain may explain why depression develops. In other words, the neurochemical, neuroendocrine, neurophysiological and neurohistological effects of stress on the brain could be the biological mediators of depression in humans who are vulnerable to the disorder.

### 2. Animal models of stress and depression are valid models for the understanding of the neurobiology of stress, depression and antidepressant action in humans

There is a great deal of literature on the effects of various forms of stress on the biology and behavior of animals and humans. Interestingly, changes in the regulation of endocrine systems, changes in learning and memory, changes in the histology of certain parts of the brain, changes in behavior and other changes show strong resemblances between stressed animals and depressed humans. This appears to validate the use of animal models to explain stress and antidepressant mechanisms in humans. Therefore, stress-induced brain changes in animal models of depression may validly represent the brain changes in depressed humans, and antidepressant-induced changes in animal models may validly represent antidepressant mechanisms in depressed humans.[4]

### 3. Stress-induced neurohistological changes are fundamental to depression

Among the various brain changes induced by stress, the neurohistological changes may be the most fundamental as an explanatory model for depression. This is because changes in neuronal architecture and connectivity would likely cause fundamental and persistent impairments in the functioning of the affected neuronal territories. Thus, the cognitive, affective and behavioral impairments in depression could result from the neurohistological changes (identified in animal models of stress and depression) that develop in the brain territories that subserve these functions.

### 4. If antidepressants produce neurohistological changes that are opposite to those resulting from stress, then such changes may explain antidepressant activity

In animal models, stress impairs neuroplasticity in certain parts of the brain and induces neuroplasticity in other parts of the brain; what these changes are and how they may impair cognition, affect and conation are discussed in later sections. In animal models, antidepressant drugs reverse many of the behavioral and other effects of stress. Furthermore, in animal models, antidepressant drugs reverse some of the stress-induced neurohistological changes with a time course that parallels the time course of antidepressant action in humans. It is therefore reasonable to speculate that antidepressant drugs reverse depression in humans by reversing the neurohistological effects of stress.

### Cortisol as a villain of the piece

Mild stress is usually associated with successful adaptation to the environment. At the neurohistological level, mild stress is also associated with the favorable neuroplasticity changes that are described later in this article. These neurohistological changes may therefore represent the biological correlates of the learning that is responsible for adaptation to the stress.

Glucocorticoid receptors are richly expressed on neurons in the hippocampus and elsewhere in the brain.[5] Mild stress may therefore act through physiological glucocorticoid signaling. Certainly, at physiological levels, the stress hormone cortisol stimulates hippocampal neurons and facilitates learning and memory. At pathological levels, however, cortisol overstimulates the hippocampal neurons and causes dendritic atrophy and loss of synapses; whereas these structural changes are generally reversible, in extreme situations, pathological overstimulation by cortisol can also result in neuronal apoptosis.[5,6] Glucocorticoid agonism in the medial prefrontal cortex also influences learning and memory: memory consolidation is facilitated, but working memory is impaired.[7]

These neurohistological effects of hypercortisolemia are seen in animal models as well as in humans. In animal models, the neurohistological changes develop in models of stress and depression as well as after chronic administration of corticosteroids to nonstressed animals. In humans, cognitive impairment and reduction in hippocampal volume are described in depression (in which disorder hypercortisolemia is known to occur), in Cushing’s syndrome as well as after the chronic administration of glucocorticoids for medical indications.[8]

Interestingly, the glucocorticoid receptor antagonist mifepristone has been suggested as a treatment for psychotic depression.[9] It may also attenuate the cognitive impairments associated with depression.[10]

## KEY BRAIN STRUCTURES AFFECTED BY STRESS

In animal models, there are at least three important brain territories in which stress induces significant neurohistological changes. These are the hippocampus, the prefrontal cortex and the amygdala.

### 1. Stress and the hippocampus

In animal models, stress-induced histological changes in the hippocampus include the following:[3,11–15]

- Loss of dendritic spines
- Decrease in the number and length of dendrites
- Loss of synapses
- Loss of glia
- Impairment of neurogenesis
- Possibly, apoptosis (under extreme conditions)

In consequence, there is a reduction in hippocampal volume; such reduction has been observed postmortem in animal models of stress and depression[3,11] as well as in magnetic resonance imaging (MRI) studies of depressed humans.[16]

Why are these histological changes important? Here is a simple explanation. There are about a hundred billion neurons in the human brain. Each neuron synapses with an average of about a thousand other neurons, making about one hundred trillion synapses in the brain.[17] Just as the social support, social importance and social effectiveness of an individual depend on his social networks, so too does the functional capacity of a neuron depend on its synaptic networks and connectivity. When dendrites and synapses are lost, this connectivity decreases, and the affected neurons become less effective in the circuits in which they lie. Loss of glia (which also play an important role in neurotransmission), decreased neurogenesis and apoptosis magnify the impairment.

The hippocampus has projections to the dorsolateral prefrontal cortex, the ventral tegmental area and the hypothalamus. Stress-induced histological changes in the hippocampus could therefore disturb not only hippocampal functioning but also functioning in these downstream areas.[3]

The hippocampus is a key structure involved in learning and memory, especially explicit (consciously acquired) memory. Perhaps as a result of hippocampal impairment, stressed animals and depressed humans show impaired learning and memory.[8,18] Insofar as coping necessitates intact learning mechanisms, hippocampal impairment could also explain compromised coping behavior observed in depression.

The dorsolateral prefrontal cortex coordinates with the hippocampus in the regulation of explicit memory. It also subserves other important cognitive functions, such as attention and concentration. These cognitive functions are also impaired in depression, perhaps as a downstream effect of hippocampal impairment.[3]

The ventral tegmental area projects to the nucleus accumbens. Mesolimbic circuitry regulates the response to novelty and the experience of reward. Stressed animals show impaired response to novelty. The parallel in depressed humans could be anhedonia. Thus, hippocampal impairment may explain anhedonia as a downstream effect.[3]

The hypothalamus regulates the autonomic nervous system and the neuroendocrine system. Disturbed hypothalamic inputs, perhaps associated with downstream hippocampal impairment, may explain some of the neuroendocrine and autonomic nervous system disturbances that characterize depression.[3]

### 2. Stress and the prefrontal cortex

In animal models, stress-induced histological changes in the prefrontal cortex include the following:[3,11,13,15]

- Loss of dendritic spines
- Atrophy of the dendritic tree
- Loss of synapses
- Decreased number and size of glia

Postmortem studies in depressed humans reveal a decrease in neuronal size, a decrease in glial size and number and a decrease in overall cortical thickness.[11]

The prefrontal cortex regulates cognitive functions such as attention, concentration, learning and memory. The prefrontal cortex also regulates higher mental functions such as motivation and judgment. All these functions are impaired in depression, perhaps as a result of the neurohistological prefrontal changes associated with stress and depression.

### 3. Stress and the amygdala

In animal models, stress-induced histological changes in the amygdala are strikingly different from those in the hippocampus and prefrontal cortex. Changes described include the following:[3,15]

- Increased dendritic arborization
- Increased synaptogenesis

The resultant increase in amygdalar volume has been described in both stressed animals and depressed humans. The increase is not merely structural; it is functional as well.[3] A recent meta-analysis of 13 MRI studies of the amygdala in unipolar depression however found that amgydalar volume increase was a function of antidepressant treatment.[19] A possible explanation for this discordant result is the varied definition of the boundaries of the amygdala in the different studies, as the amygdala is not a clearly demarcated structure.[12]

The amygdala is involved with social and emotional learning and, especially, with emotions such as anxiety and fear.[12] Fear learning is upregulated in stressed animals, i.e., they show an exaggerated, persistent and more generalized fear response to anxiogenic and noxious stimuli.[3] As a parallel, depressed humans are often anxious and afraid and show an upregulated anxiety response to minor provocations.

Interestingly, animal research shows that whereas stress-induced changes in the hippocampus gradually reverse after the removal of the stress, stress-induced changes in the amygdala do not reverse for weeks or longer.[3] In fact, the persistence of amygdalar changes may explain why depressed humans overreact to stress in a trait-dependent way, why current depression begets future depression, why life events have a cumulative effect in the predisposition to depression and even why physical and sexual abuse of children predisposes to depression during adult life.

### What neurohistological changes explain the symptom of depression?

The preceding discussion explains why anxiety, fear, anhedonia, motivational impairment, cognitive deficits, neuroendocrine changes and autonomic nervous system dysfunction may occur in the context of the neurohistological changes induced by stress. It does not explain how or why depression as a symptom develops.

Perhaps the best way to address this challenge is to consider that no single neuroanatomical locus explains depression, no more than a single locus in the brain explains happiness or other complex emotions. In other words, depression, like schizophrenia, could be the sum total of disturbances in the functioning of multiple parts of the brain. That is, functional changes in different parts of the brain give rise to neuroendocrine, neurovegetative, neurocognitive, neurobehavioral and other deficits, which, put together, form the depressive syndrome. Whereas depression as a specific symptom is subjective and cannot be captured in animal models, it can be inferred from animal behavior, much as anxiety and fear can be inferred from animal behavior; and the neuroendocrine, neurovegetative, neurocognitive, neurobehavioral and other deficits associated with depression can individually be studied in animal models, as also the brain territories that are implicated in the genesis of these deficits.

### Why did natural selection favor an aberrant neurohistological response to stress?

Anxiety and fear as a result of amygdalar hyperactivity would, presumably, program the organism to avoid future exposure to potential stressors. This could have survival value for the organism and hence the species, and would therefore favor evolutionary selection. Downregulating the hippocampal and prefrontal response could be useful in this regard because, for survival value, an organism would need to react quickly to avoid stress rather than to think about the stress, analyze it and exhibit a reasoned response.

In present day humans, however, while the amygdalar response to stress could still have value, the aberrant hippocampal and prefrontal response may be an evolutionary dead weight. Arguments for the evolutionary survival value of depression have been expressed elsewhere.[20]

### What Antidepressants Do: The Nuts and Bolts of Neuroplasticity.

Clinicians are familiar with the concept of neuroplasticity to the extent that it explains how one part of the brain may take over some or most of the functions of another, usually adjacent, part if that part suffers damage due to causes such as trauma or ischemia. Neuroplasticity is now more broadly conceptualized as the ability of elements in the brain to exhibit structural and functional changes in response to external or internal perturbations.

At the neuronal level, neuroplasticity as a consequence of chronic treatment with antidepressant drugs more specifically refers to the cascade of neurophysiological, neurochemical and neurohistological changes that result in synaptic strengthening, dendritic growth and branching and new synapse formation. Importantly, neurogenesis, gliogenesis, enhancement of vascular endothelial support and inhibition of apoptotic mechanisms have also been described, especially in the context of ECT. Whereas most of the literature focuses on the development of these changes in the hippocampus, similar changes have been described in the prefrontal cortex as well. However, neurogenesis as a response to antidepressant treatment does not occur outside the hippocampus.

Neuroplasticity changes classically described in the hippocampus in the context of learning and memory are the same as those resulting with chronic antidepressant treatment;[3] the significance of this observation will be discussed later. Early and late neuroplasticity changes are summarized in the sections that follow. Readers may note that these changes are common to a wide range of antidepressant drugs and treatments, as will also be emphasized in a later section.

### 1. Neuroplasticity – Early changes

Neuroplasticity is initiated by the excitatory neurotransmitter glutamate. Glutamate binds to the postsynaptic NMDA receptor, which gates the calcium ion channel. Entry of calcium into the postsynaptic neuron initiates a series of changes; these involve neurotransmitter and neuromodulator molecules such as arachidonic acid, prostanoids, endogenous cannabinoids, platelet activating factor and others. The activation of these lipid-signaling pathways is ramped up by positive-feedback reverberating circuits that involve retrograde neurotransmission; it is regulated by enzymes such as COX-2; and it is dampened by endogenous chemicals such as kynurenic acid.[21]

NMDA activation also results in the activation of calcium calmodulin-dependent kinase II (CaMKII), in an increase in the number of AMPA receptors (like the NMDA receptor, the AMPA receptor is also an ionotropic glutamatergic receptor) and in an activation of silent synapses.[3]

The immediate end result of the glutamatergic cascade is long-term potentiation (LTP). LTP is the synaptic strengthening that represents the occurrence of learning and memory. Long-term depression (LTD) is also described; this is synaptic weakening that presumably represents weakening of memories or unlearning. It is important to understand here that learning and memory need not necessarily refer to an explicit target; it may also address neurocircuitry involved in healthy or unhealthy cognitions, mood states and behavior. Readers are referred to Mitchell and Baker[22] for an update on the role of glutamate in depression.

### 2. Neuroplasticity - Long-term changes

Neuroplasticity is more than just a reprogramming of synapses in the form of LTP and LTD. It involves changes in neuronal hardwiring as well. These changes are mediated by the induction of neurotrophic factors such as brain-derived neurotrophic factor (BDNF), which acts on tyrosine kinase (TrK) receptors to activate intracellular cascades involving cAMP-dependent protein kinase A (PKA), mitogen-activated protein kinase (MAPK), CaMKII and others. These chemicals activate transcription factors such as cAMP-responsive element binding protein (CREB), which in turn activate the synthesis of enzymes and proteins that are involved in the structural expression of neuroplasticity. Examples of important genes that are induced include BDNF, vascular endothelial growth factor (VGEF) and others. CREB and BDNF are among the most important players examined in neuroplasticity research.[3,11]

The end result of these intracellular signaling cascades is a stimulation of neurogenesis in the dentate gyrus. In both hippocampus and prefrontal cortex, there is also an increase in glial cells, an increase in the richness and complexity of dendritic branching and an increase in the formation of new synaptic connections. The time course of these neuroplasticity changes parallels the time course of antidepressant action, making it reasonable to infer that these antidepressant-induced neurohistological effects are responsible for the initiation of clinical recovery.[3] Interestingly, evidence of antidepressant-induced structural brain changes may be detectable in MRI scans. For example, recently, Malykhin *et al*,[16] observed increased hippocampal body volume in medicated patients with major depressive disorder relative to unmedicated patients.

Why might the induction of neuroplasticity predispose to an antidepressant response? A reasonable hypothesis is that it reverses the histological and, hence, the cognitive, behavioral and other impairments induced by stress; these impairments were described in earlier sections.

Whereas antidepressant drugs reverse the structural and functional consequences of stress in the hippocampus and prefrontal cortex, they do not reverse the changes observed in the amygdala.[18] The vulnerability to stress therefore remains, providing a biological explanation for the observed need for maintenance antidepressant therapy after the successful treatment of depression with medication. Importantly, antidepressant treatments prevent many of the neurohistological changes caused by stress[3,11] and this may explain how and why antidepressants protect against stress-induced relapse into depression.

### Learning, memory and antidepressant-induced neuroplasticity

There is a striking similarity in the neuroplasticity changes induced by antidepressant drugs and the changes that underlie learning and memory. As these neurohistological changes are in the opposite direction to the changes resulting from stress and depression, it is reasonable to speculate that antidepressant treatment is therapeutic through an induction of learning and memory mechanisms at neurochemical and neurohistological levels.

Why should the induction of learning and memory mechanisms assist or favor the recovery from depression? Speculatively, neuroplasticity may provide the wherewithal for the hardwiring of healthier cognitions, affective responses and behavioral expressions through the learning of healthier cognitive strategies and coping methods. Such adaptative learning may be facilitated by psychotherapy, or may merely arise through exposure to a healthy and supportive environment.

### Neuroplasticity as a common mechanism of antidepressant action

Antidepressant drugs are not the only treatments that induce neuroplasticity. There is a robust body of literature that shows that ECT induces similar neuroplasticity changes;[23–28] in fact, ECT may be the most potent inducer of neuroplasticity in the brain. Physical exercise, which has been reported to have antidepressant effects, also induces neuroplasticity changes.[29–30] Presumably, to the extent to which CBT requires learning and adaptation, neuroplasticity may be said to underlie CBT as well. Finally, data also document neuroplasticity with other treatments that have antidepressant effects, ranging from quetiapine[31,32] to tDCS.[33] Neuroplasticity would therefore seem to be a final common pathway or a grand unifying mechanism of action of different antidepressant therapies.

### Other notes

Neurohistological changes in stress and antidepressant response are site specific. For example, as already described, stress results in opposite structural and functional changes in the amygdala as compared with the hippocampus and prefrontal cortex. Then, whereas increased expression of CREB and BDNF in the hippocampus reverses stress-induced changes in this structure, increased expression of CREB and BDNF in the nucleus accumbens increases learned helplessness and social defeat in animal models of depression.[3]

Future treatments for depression may directly attempt the induction of neuroplastic changes. For example, the intraventricular administration of BDNF has antidepressant effects in animal models. The induction of the cAMP-CREB cascade by rolipram reverses animal models of depression but, unfortunately, has had unimpressive effects in clinical settings.[3]

## CONCLUDING OBSERVATIONS AND SPECULATIONS

Is the neuroplasticity hypothesis of antidepressant action consistent with findings in clinical or research settings? Some observations are briefly examined and speculative reconciliatory responses are provided. These can be the basis for future research in the field.

*Observations*: Episodes of depression may occur in the apparent absence of stress. Stress does not necessarily trigger a depressive state. Some individuals seem to thrive on stress. Stress may result in different forms of depression: grief, dysthymia or major depressive episodes. Stress may result in disorders other than depression.

*Reconciliation*: There is no such thing as an absence of stress. In fact, the very stresses and strains of everyday life may be neurotoxic to vulnerable individuals, with vulnerability defined by genetic and epigenetic mechanisms. Furthermore, as life event research has shown, episodes of illness can be triggered even by positive or desirable life events. Genetic mechanisms may explain why stress has no harmful effect in some individuals but triggers depression in others. Genetic mechanisms may also explain why different individuals experience different forms of depression, and why some individuals suffer depression whereas others develop schizophrenia or other psychiatric disorders in response to stress. Of note, neuroplasticity with antidepressants would not be expected to relieve the primary symptoms of schizophrenia because schizophrenia is histologically far different from depression.

*Observation*: Each episode of depression increases the chances that subsequent episodes will be more likely, more frequent, more severe, longer lasting and more difficult to treat.

*Speculation*: Antidepressant-induced neuroplasticity may not completely reverse the neurotoxic effects of stress. As a result, long-term structural changes in the hippocampus and prefrontal cortex may chronically compromise coping behavior. Additionally, amygdalar overlearning may prevent the extinction of unhealthy emotional reactions to stressful situations, which may have a further negative impact on the course of illness. The situation may be analogous to a weak knee; once damaged, it is weak for life.

*Observation*: The benefits of the antidepressant-CBT combination are generally greater than the benefits of antidepressant treatment alone.

*Speculation*: CBT may help imprint healthy cognitions and behaviors on the neuroplasticity changes induced by medication.

*Observation*: Some patients do not respond to antidepressant drugs.

*Speculation*: There may be a critical period for imprinting healthy cognitions and behaviors after the initiation of antidepressant therapy. If unfavorable environmental circumstances do not permit the establishment of healthy neurocircuitry during this critical period, antidepressant refractoriness may result. The continuation of stress may antagonize the action of antidepressant drugs at the neurohistological level; this may also explain antidepressant resistance. Finally, genetic and epigenetic mechanisms may also play a role in treatment resistance.

*Observation*: Some forms of depression, such as dysthymia and the grief reaction, are less responsive to antidepressant drugs than major depressive episodes.

*Speculation*: Dysthymia is characterized by a deeply ingrained failure to adapt to the stresses and strains of everyday life. Mere induction of neuroplasticity may not suffice to treat dysthymia; the imprinting of improved coping mechanisms (through appropriate psychotherapy) on the newly formed neurocircuits may be necessary. With regard to the grief reaction, affected individuals may not have an inherent genetic vulnerability to necessitate antidepressant medication for the adaptation to the stress. It may be noted here that all individuals have an innate neuroplastic capability, and it may suffice for some depressed individuals to merely learn adaptative behaviors to recover from depression; antidepressant drugs may not always be necessary.

*Observation*: Antidepressants are often no better than placebo in clinical trials conducted in depressed children and adolescents.

*Speculation*: Children have an intrinsically plastic brain and may not need antidepressants as much as adults may. Alternately, children may lack the maturity to utilize the neuroplastic changes toward an emotionally and psychosocially adaptative response.

*Observation*: Different antidepressant drugs are associated with different degrees of efficacy in different patients.

*Speculation*: Different antidepressant drugs have different effects on the pathways that mediate the neuroplasticity response, and different patients have different genetic and epigenetic reasons for their depression. These variations may explain differences in antidepressant efficacy in different patients.

*Observation*: Antidepressant drugs are effective in disorders other than depression.

*Reconciliation*: The efficacy of antidepressant drugs in different psychiatric disorders may require the induction of neuroplasticity only if these disorders are also due to faulty learning and can be corrected by unlearning and relearning. Antidepressant drugs may also act through nonneuroplasticity mechanisms. For example, local anticholinergic actions of the TCA may suffice for benefits in acid-peptic disease or the irritable bowel syndrome.

## SUMMARY

Stress and depression result in loss of dendritic spines, dendritic atrophy and loss of synapses in the hippocampus and prefrontal cortex; glial cells decrease in number and size. In consequence, hippocampal and prefrontal functioning are impaired, as also the functioning of downstream structures such as the nucleus accumbens and hypothalamus. In contrast, stress is also associated with increased dendritic arborization and new synapse formation in the amygdala; the functioning of this structure may be amplified. The neurohistological changes in these brain territories may explain why anhedonia, amotivation, anxiety, fear, cognitive deficits, neuroendocrine changes and autonomic nervous system changes characterize depression.

Antidepressant drugs are associated with the induction of neuroplasticity in structures such as the hippocampus and prefrontal cortex: there is stimulation of neurogenesis, gliogenesis, dendritic arborization and new synapse formation. These changes may underlie the mechanisms of antidepressant response because their time course of development parallels the time course of antidepressant action, because they reverse the neurohistological effects of stress and because they may allow the relearning of healthier cognitions, healthier emotional responses and healthier behavioral expressions.

The neuroplasticity hypothesis for antidepressant action is by no means certain or complete. For example, it does not explain why ketamine,[34–35] scopolamine[36–37] and ECT[38] may have dramatic antidepressant effects. However, it is far more plausible and complete than any of the earlier hypotheses of antidepressant action.

### Recommended reading

This article, as the title indicates, is a primer on stress, depression, antidepressant treatment and neuroplasticity. There have been several excellent technical reviews published on the subject during the past decade, and the reader is referred to these reviews for an elaboration on the subject.[3,11,39]
